# A physiologically based pharmacokinetic model to optimize the dosage regimen and withdrawal time of cefquinome in pigs

**DOI:** 10.1371/journal.pcbi.1011331

**Published:** 2023-08-16

**Authors:** Kun Mi, Lei Sun, Yixuan Hou, Xin Cai, Kaixiang Zhou, Wenjin Ma, Xiangyue Xu, Yuanhu Pan, Zhenli Liu, Lingli Huang

**Affiliations:** 1 National Reference Laboratory of Veterinary Drug Residues (HZAU) and National Safety Laboratory of Veterinary Drug (HZAU), Wuhan, China; 2 MOA Key Laboratory for Detection of Veterinary Drug Residues, Wuhan, China; 3 MOA Laboratory for Risk Assessment of Quality and Safety of Livestock and Poultry Products, Wuhan, China; 4 College of Veterinary Medicine, Huazhong Agricultural University, Wuhan, China; University at Buffalo - The State University of New York, UNITED STATES

## Abstract

Cefquinome is widely used to treat respiratory tract diseases of swine. While extra-label dosages of cefquinome could improve clinical efficacy, they might lead to excessively high residues in animal-derived food. In this study, a physiologically based pharmacokinetic (PBPK) model was calibrated based on the published data and a microdialysis experiment to assess the dosage efficiency and food safety. For the microdialysis experiment, *in vitro*/*in vivo* relative recovery and concentration-time curves of cefquinome in the lung interstitium were investigated. This PBPK model is available to predict the drug concentrations in the muscle, kidney, liver, plasma, and lung interstitial fluid. Concentration-time curves of 1000 virtual animals in different tissues were simulated by applying sensitivity and Monte Carlo analyses. By integrating pharmacokinetic/pharmacodynamic target parameters, cefquinome delivered at 3–5 mg/kg twice daily is advised for the effective control of respiratory tract infections of nursery pig, which the bodyweight is around 25 kg. Based on the predicted cefquinome concentrations in edible tissues, the withdrawal interval is 2 and 3 days for label and the extra-label doses, respectively. This study provides a useful tool to optimize the dosage regimen of cefquinome against respiratory tract infections and predicts the concentration of cefquinome residues in edible tissues. This information would be helpful to improve the food safety and guide rational drug usage.

## Author summary

Physiologically-based pharmacokinetic (PBPK) models, a mechanism-based model that incorporate physiological and chemical-specific parameters, can describe the absorption, distribution, metabolism and excretion of chemicals in different organs of animals. It can be applied for chemical risk assessment, dosage optimization and withdrawal intervals determination. The Extra-label use antimicrobial drug is a way to solve the treatment failure resulting from antimicrobial resistance. Integrate with pharmacokinetic/pharmacodynamic (PK/PD) parameters and maximum residual limit, PBPK model can determine a safe and effective dosage. This approach would be helpful to improve the food safety and guide rational drug usage.

## Introduction

Cefquinome (CEQ), a fourth generation of cephalosporins, is solely developed for veterinary use. CEQ can interrupt cell wall formation and exert excellent antibacterial effects on both gram-positive and gram-negative bacteria *in vivo* and *in vitro*. CEQ has been approved to treat respiratory tract infections of swine by intramuscular administration of 1–2 mg/kg bodyweight for 3–5 days [1]. *Actinobacillus pleuropneumoniae* (APP), *Haemophilus parasuis*, *Pasteurella multocida*, and *Streptococcus suis* are the main infectious microorganisms for swine respiratory tract diseases [2]. A myriad of antibacterial drugs, including CEQ, are gradually losing their antibacterial efficacy [3] because of drug resistance caused by long-term heavy use. To prevent the emergence of resistance and to improve the effects of CEQ against respiratory tract diseases, it is essential to optimize the dosage regimen. However, the optimized extra-label dosage regimen may lead to excessive concentration of residues in tissues that violate regulations [4]. Hence, it is necessary to develop a method to optimize the dosage regimens of CEQ for different animal groups, and to predict the drug residues and the withdrawal intervals (WDIs) for different dosage regimens.

A physiologically based pharmacokinetic (PBPK) model can simulate the absorption, distribution, metabolism, and excretion of drugs in the body because it is established based on physiology, anatomy, and compound specificity [5]. PBPK models have been used to predict the concentration of veterinary antibacterial drugs in different tissues [6,7]. Diffusion-limited PBPK models can be applied to predict the drug concentration in the interstitial fluid and cells of organs, which are thought to comprise blood, interstitial fluid, and tissue (cell) sub-compartments. Researchers have developed it to predict the florfenicol concentration in lung interstitial fluid [8]. A PBPK model was model developed to predict the toxicology of gold nanoparticles for different organ-specific cells [9].

The biophase for extracellular pathogens is the pulmonary epithelial lining fluid, and swine respiratory tract pathogens can survive in the lung interstitial fluid. It is essential to explore the pharmacokinetic (PK) profiles of unbound drugs in the target tissue. Microdialysis is a gold standard to measure the unbound antibacterial drug in lung interstitial fluid [10]. By integrating microdialysis, a PBPK model is capable of predicting the drug concentration in the lung interstitial fluid. The PK/pharmacodynamic (PD) parameters of lung interstitial fluid in virtual individuals are determined by comparison with target values, allowing the dosage to be optimized [11,12]. The probability of target attainment (PTA) is the percentage of individuals reaching the target PK/PD parameters at different bacterial minimum inhibitory concentrations (MICs). This information has been used to support and justify dose recommendations across populations [13]. Monte Carlo analysis (MC) can be incorporated into the PBPK model to establish a population PBPK (pop-PBPK) model that can address the interindividual variability and predict PK profiles. According to published guidance [14], a pop-PBPK model is use to estimate the WDI. Indeed, pop-PBPK models have been widely used to determine WDIs while considering maximum residue limits (MRLs) [15] and toxicological assessment [16].

This study aimed to establish a PBPK model to predict the unbound CEQ concentration in lung interstitial fluid and edible tissues based on a PK microdialysis investigation of CEQ in lung interstitial fluid. After performing microdialysis, the PD parameters of CEQ against different swine respiratory tract pathogens and MC were integrated to optimize the dosage regimen and predict the WDIs of different CEQ dosage regimens in pigs. The findings from this investigation should improve the scientific application of CEQ and ensure food safety.

## Results

### *In Vitro*/*In Vivo* relative recovery

**Fig 1:**
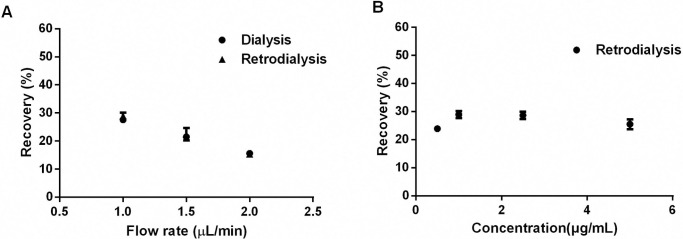
**Influence of the cefquinome concentration (A) and perfusion fluid flow rate (B) on *in vitro* recovery determined by microdialysis and retrodialysis.** The data are presented as the mean ± standard deviation.

**Fig 1** shows the influence of flow rates and drug concentrations on recovery. The flow rate of 1 μL/min had the best *in vitro* relative recovery (RR) (27.59% ± 0.10%). At the flow rate, the delivery (%) was steady (CV < 11%). The *in vitro* RR of microdialysis and retrodialysis was similar for different flow rates, a finding that indicates retrodialysis can be adopted to investigate the *in vivo* RR. After probe acclimatization, a sample with 0.75 μg/mL CEQ was driven by the pump as flow rate of 1 μL/min. The dialysate was collected every 15 min. Based on Eq 3, the *in vivo* RR was 28.8% ± 2.74%.

### PK profile of CEQ in the dialysate

**Fig 2:**
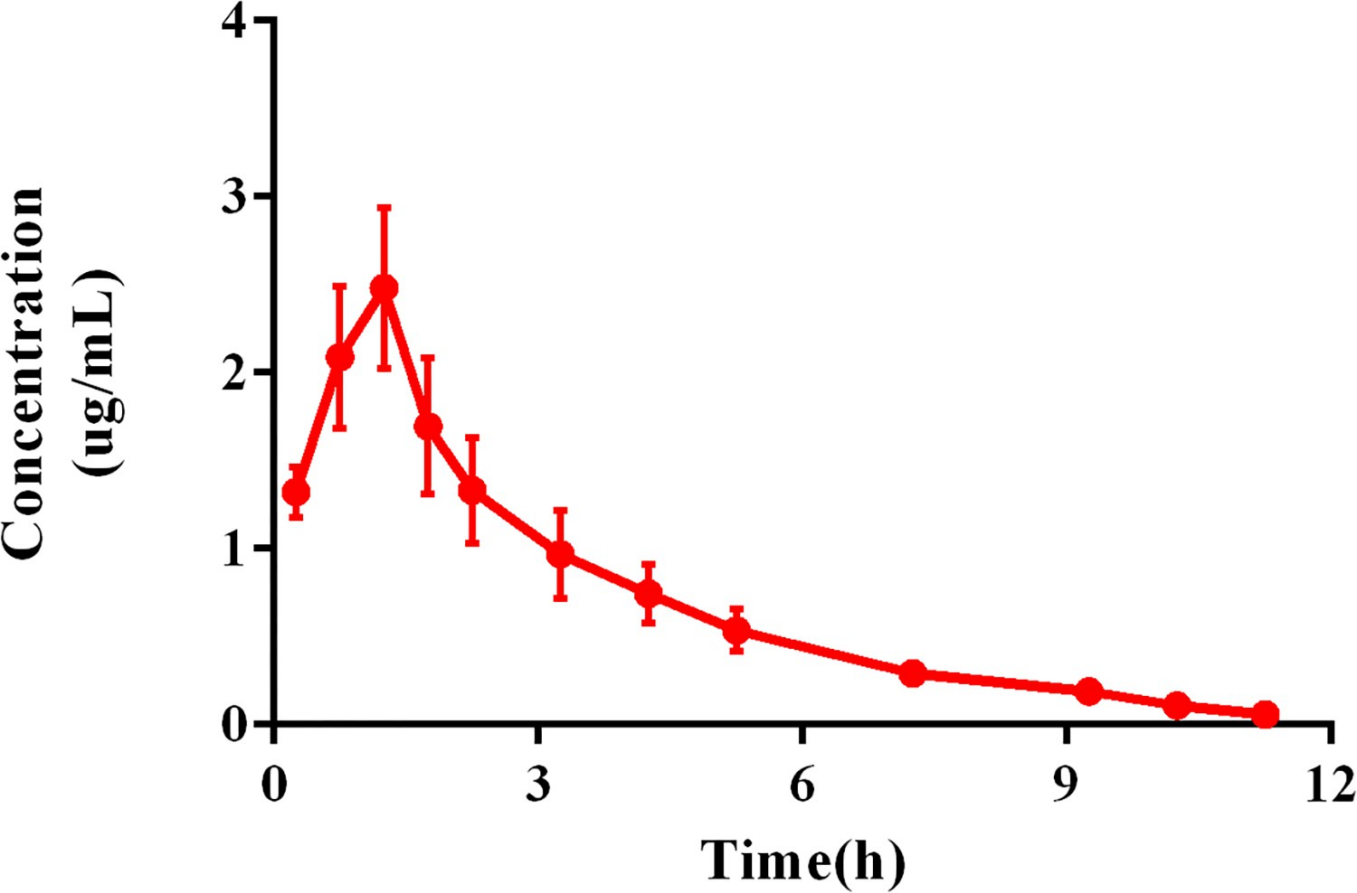
Concentration-time profile of cefquinome in the dialysate. The data are presented as the mean ± standard deviation (n = 4).

The PK parameters of CEQ in dialysate were calculated by Phoenix version 8.3. CEQ was absorbed into the lung and reached the maximum concentration of 2.48 ± 0.46 μg/ml at 1.25 h. The elimination half time (T_1/2 λ_) was 1.34 ± 0.38 h, and the mean residence time was 3.08 ± 0.15 h. The AUC of the concentration-time curve was 8.40 ± 1.62 μg*h/mL. Integrated with the previous study reported that AUC_plasma_ is 9.77 ± 0.63 μg*h/mL [17], the ratio of AUC_lung interstitial fluid_ to AUC_plasma_ was 0.86. Concentration-time profile of cefquinome in dialysate was shown in **Fig 2.**

### PBPK model calibration

**Fig 3:**
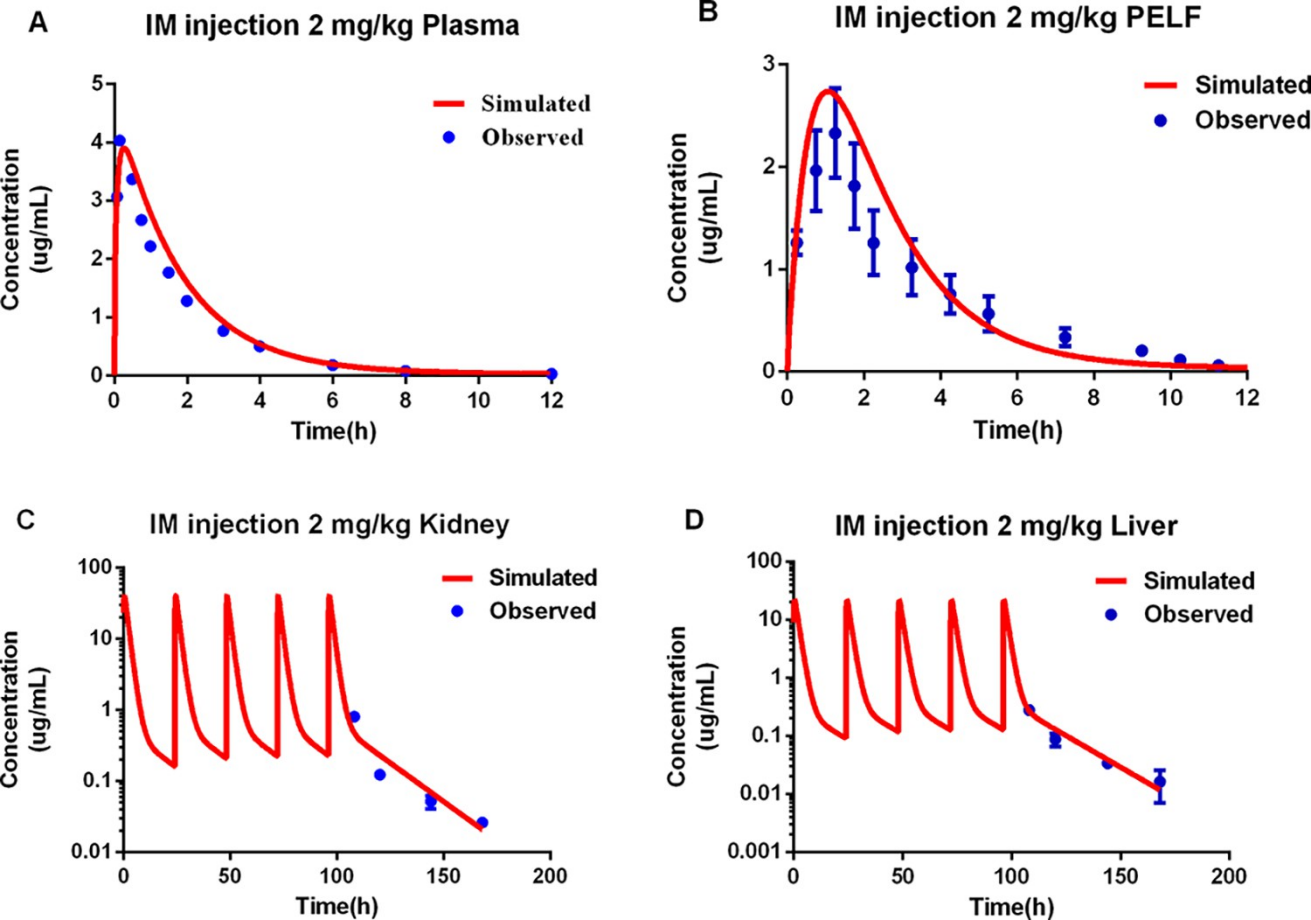
Calibration of the physiologically based pharmacokinetic model. Comparison of the model simulation (red line) and the observations (blue circles) for the cefquinome concentration in the plasma (A), lung interstitial fluid (B), kidney (C), and liver (D). For (C) and (D), cefquinome was delivered via five intramuscular doses of 2 mg/kg and the observed data are from Zhang, Li [18]. The obsreved data in plasma are from Li, Wu [19]. And the observered data in lung interstitial fluid are determined by this manuscript.

The predicted CEQ concentrations in the plasma, edible tissues, and lung interstitial fluid at different time points were compared with the observed data and were shown in **Fig 3**. Overall, the model provided a good simulation of the kinetic profile in different tissues. For the repeated-dose scenarios in the kidney and liver, at the later time points the model could accurately predict the CEQ tissue concentrations.

### Model validation

**Fig 4:**
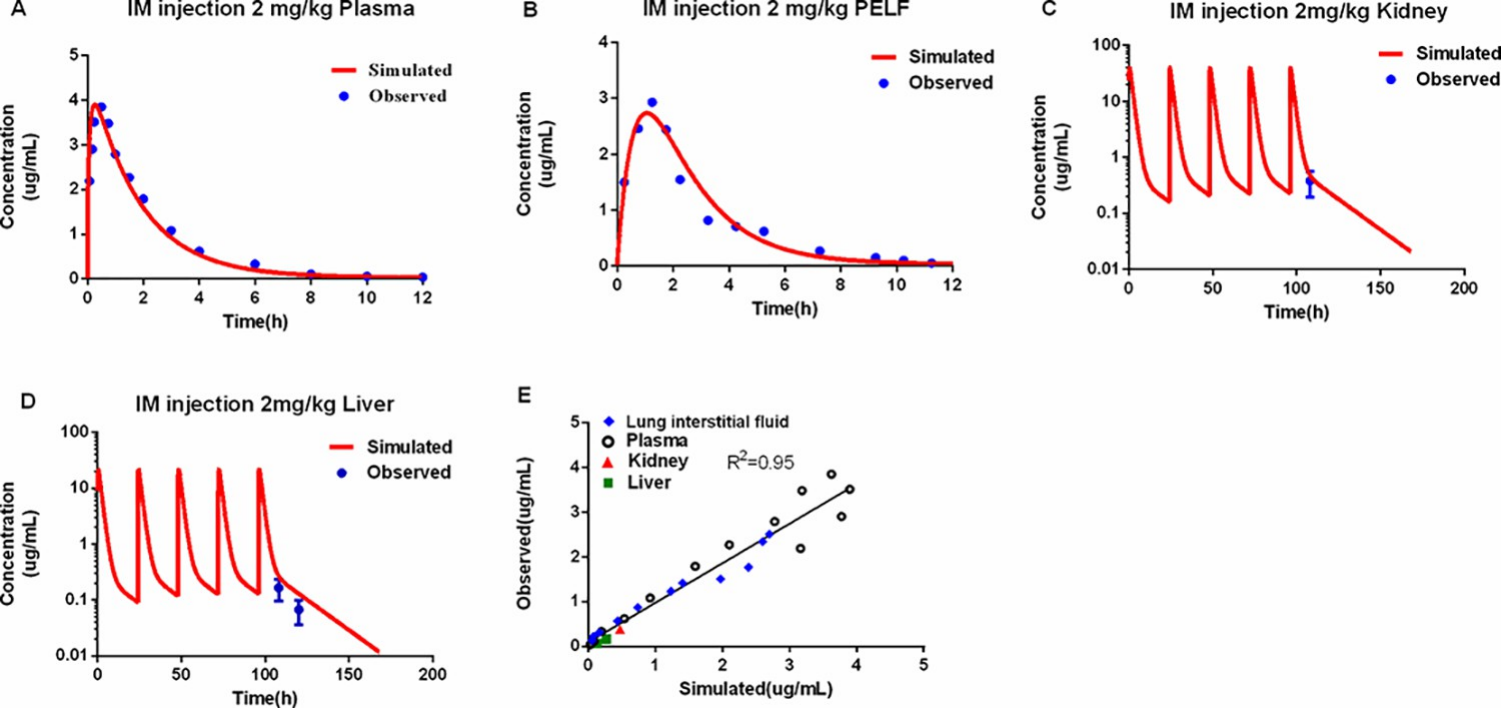
Validation and regression analysis of the physiologically based pharmacokinetic model. Comparison of the model simulation (red line) and the observed data (blue circles) in plasma (A), lung interstitial fluid (B), kidney (C), and liver (D). Regression analysis (E) was performed between the simulation and the observations (R^2^ = 0.95) the observed data liver and kidney are from Xu., Yang. [21]. The obsreved data in plasma are from Mi, Li [17]. And the observered data in lung interstitial fluid are determined by this manuscript.

The dataset for model calibration and validation are different. The PBPK model could accurately predict data at different time points. The observed CEQ concentrations in the plasma after a single 2 mg/kg intramuscular injection and in edible tissues five daily 2 mg/kg intramuscular injections were compared with the simulated results (**Fig 4**). The goodness of fit was evaluated by R^2^ between the measured and simulated CEQ concentrations in plasma and edible tissues. The R^2^ of 0.95 is acceptable (≥ 0.75; [20]). The calculated MAPE ranged from 15.87% to 41.90% (**S1 Fig**), an acceptable result. Overall, the PBPK model adequately captured the kinetic profiles of CEQ in relevant edible tissues, plasma, and lung interstitial fluid.

### Sensitivity analysis

A local sensitivity analysis was carried out for 31 parameters. A parameter was considered to be influential if the absolute value of its NSC was > 0.25 [15]. The local sensitivity analysis was based on 10% variation for the AUCs of different compartments (**S2 Table**). For the physiological parameters, cardiac output (QCC) and kidney flow rate (QKC) are sensitive parameters for the present model. These physiological parameters are not sensitive to the other parameters in this model. The partition coefficients P_liver_, P_kidney_, and P_lung_ only have a positive impact on the AUC of their respective tissues. Renal clearance (KurineC) is highly sensitive to the AUCs of plasma, liver, kidney, and lung. For the AUC of lung interstitial fluid, except for the physiological parameters, it is sensitive to the transfer constant from interstitial fluid to tissue (KTI) and from tissue to the interstitial fluid (KIT), and lung tissue protein binding rate (PT) with NSC values of 0.70–0.67, and -0.31 respectively.

### Determination of the dosage regimen integrated with PK/PD parameters

PK/PD parameter, *f*AUC/MIC and %*f* T>MIC, is related to antibacterial effect. And it is recognized by CLSI and EMA to determine and optimize the dosage. %*f*T>MIC is the best PK/PD parameter related to cefquinome antibacterial effect.

*S*. *suis* is a gram-positive pathogen, and *H*. *parasuis*, *P*. *multocida*, and APP are gram-negative pathogens of the swine respiratory tract. %T>MIC is defined as 25%-40% of the dosage regimen for gram-positive pathogens and 40%-50% for gram-negative pathogens [22]. The 10th percentile, 50th percentile and 90th percentile under different dosages for gram-negative and gram-positive pathogens are shown in **Table 1**.

**Table 1:** The percentiles of different dosage regimens in plasma and PELF with MIC of 0.25 μg/mL and 1 μg/mL.

Dose	Tissue	MIC = 0.25μg/mL			MIC = 1μg/mL		
		10th percentile	50th percentile	90th percentile	10th percentile	50th percentile	90th percentile
2 mg/kg	Plasma^A^	15.30%	19.30%	24.10%	8.1%	10.1%	12.6%
	PELF^A^	17.60%	22.10%	27.80%	9.0%	13.4%	18.0%
	Plasma^B^	30.60%	38.70%	49.10%	16.3%	20.3%	25.4%
	PELF^B^	35.20%	45.00%	57.30%	18.6%	27.1%	35.7%
3 mg/kg	Plasma^A^	17.50%	22.30%	28.30%	10.1%	12.8%	16.2%
	PELF^A^	20.20%	25.20%	32.00%	12.5%	16.6%	21.6%
	Plasma^B^	35.50%	44.90%	56.70%	20.4%	25.8%	32.9%
	PELF^B^	40.60%	51.20%	64.60%	24.8%	33.6%	43.3%
4 mg/kg	Plasma^A^	19.30%	24.40%	31.20%	11.5%	14.5%	18.2%
	PELF^A^	22.00%	27.30%	34.10%	14.2%	18.5%	23.7%
	Plasma^B^	39.10%	49.90%	63.10%	23.2%	29.7%	37.3%
	PELF^B^	44.30%	55.30%	72.20%	28.4%	37.6%	47.8%
5 mg/kg	Plasma^A^	20.60%	26.40%	33.40%	12.7%	16.1%	20.1%
	PELF^A^	24.20%	31.00%	40.10%	15.7%	20.1%	25.7%
	Plasma^B^	41.40%	53.50%	69.70%	26.2%	32.6%	40.9%
	PELF^B^	49.4%	63.0%	85.3%	31.3%	40.6%	51.6%

Note: A is once administration daily; B is twice administration daily

With the label dose (2 mg/kg once a day), the 90th percentile of T>MIC simulation as 24.10% in the plasma and 27.80% in the lung interstitial fluid with a MIC of 0.25 μg/mL, and 12.6% in the plasma and 18.0% in the lung interstitial fluid with a MIC of 1 μg/mL. These values indicate that the label dose does not provide an effective antibacterial effect against gram-negative and gram-positive pathogens. Among the once-daily dosage regimens, 4 mg/kg seems could inhibit gram-negative pathogens in the lung interstitial fluid and the 50th percentile of simulation was 27.30% that above the PK/PD target parameter (25%). For the dosage regimen of 3 mg/kg twice daily, the 10th percentile of simulation is above the criterion for gram-negative pathogens (25%-40%). For *S*.*suis*(MIC = 1μg/mL), in PELF, the 90th percentile of simulation can reach the criteria (40%-50%). The dosage regimen of 3 mg/kg twice daily is a conservative regimen against swine respiratory tract diseases. For the dosage regimen of 5 mg/kg twice daily, the 50th percentile of simulation in PELF is above the PK/PD target parameters for both gram-negative and gram-positive which indicate a high likelihood of success.

### WDI estimation

**Fig 5:**
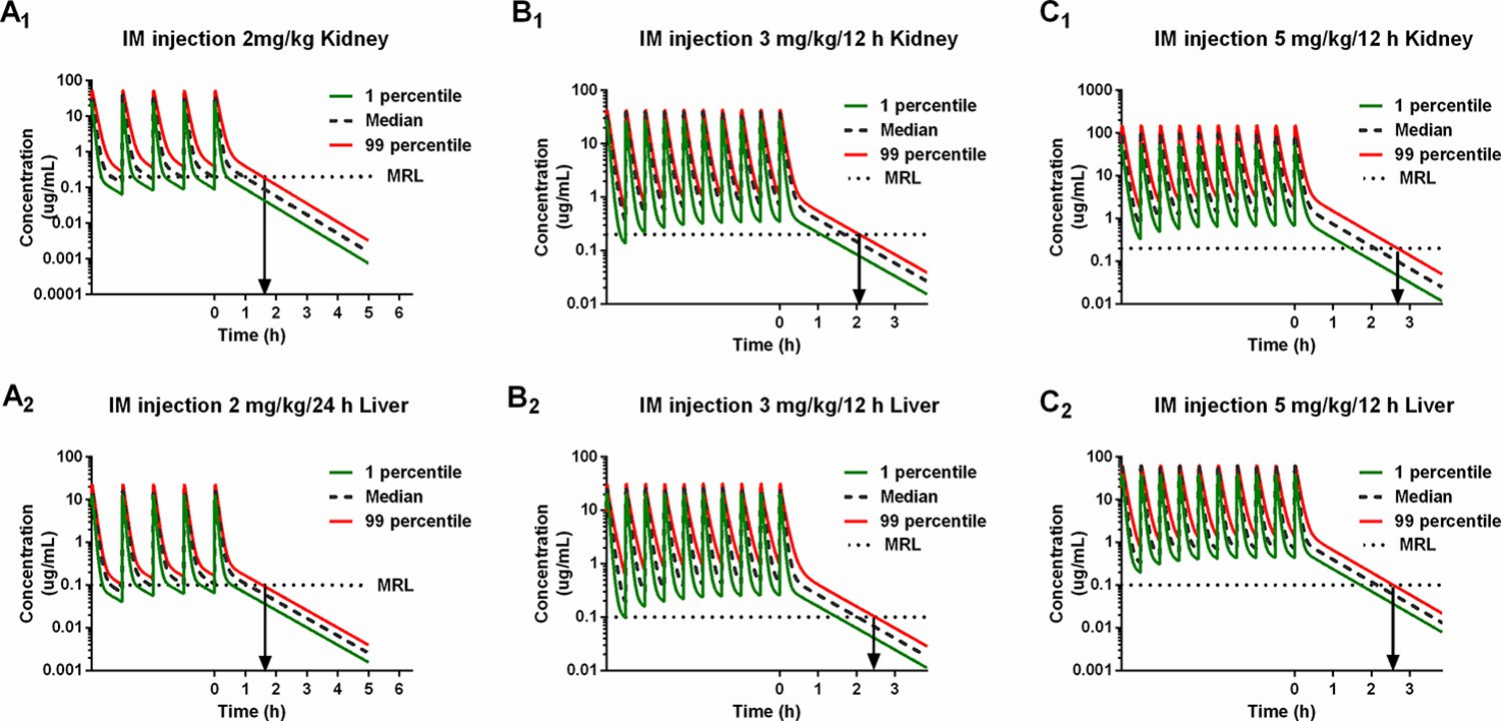
Monte Carlo analysis of cefquinome in the liver and kidney based on the population physiologically based pharmacokinetic model. The liver and kidney residue concentrations after daily injection of the label dose (2 mg/kg) or twice-daily injection of the extra-label doses (3 mg/kg and 5 mg/kg) were simulated. The median, 1st, and 99th percentiles of the simulated results were plotted. The maximum residual limitation (MRL) was 0.1 and 0.2 μg/mL in kidney and liver, respectively.

The drug level in liver and kidney are higher than other tissues. Therefore, the liver and kidney were chosen as the target tissues to determine the WDIs for the label and extra-label dosage regimens. China and EU recommend the same MRLs of 0.1 PPM in liver and 0.2 PPM in kidney [23]. As shown in **Fig 5**, the WDI for five daily injections of the label dose (2 mg/kg) is 2 days. The WDI for five twice-daily injections of the extra-label doses (3 or 5 mg/kg) is 3 days.

## Discussion

In this study, a PBPK model was established for intramuscular administration of CEQ in swine. The PBPK model was calibrated with published PK data, residue elimination data, and microdialysis data. Furthermore, the microdialysis data were used to construct a lung interstitial fluid sub-compartment. The present PBPK model integrates Monte Carlo analysis and could be used to predict the dosage regimen and withdrawal time.

In recent decades, PBPK models have contributed to the assessment of food safety [4], to determine dose-response relationships [24], and to assist in drug development and research [25]. PBPK models can be applied to predict the drug level in different tissues, even in the sub-compartments—for example, interstitial fluid. Viel et al. explored the distribution and excretion of colistin with drug concentration in different sub-compartments of the kidney by a whole-body PBPK model [26]. In addition, when combined with PK/PD models, the dose can be predicted [27]. Zhou et al. determined the dosage regimen of enrofloxacin against pathogens in the intestinal tract with the PBPK model and PK/PD parameters [28].

The physiological parameters of the current model [29] and the physiological parameters related to lung tissues [8,30] were obtained from the literature. The initial partition coefficients were assumed to be equal to the value previously reported in mice [30]. The partition coefficients were subsequently adjusted by comparing the concentration-time profiles of different tissues in swine. The parameter related to intramuscular injection indicates that CEQ was quickly absorbed into the venous circulation after administration. Kdiss, Frac, and Kim were optimized as 0.05, 0.1 and 7 per h, respectively. The elimination parameters KurineC and KblieC were optimized as 0.3 and 0.01 L/h/kg, respectively; these values are the same as reported previously [19]. For the lung interstitial fluid sub-compartment, only free drug can cross between different sub-compartments; this value was derived from the drug concentration. PT was defined as 0.3 for the final model. KBI, KIB, KIT, and KTI were optimized as 0.110, 0.052, 3.56, and 2.60 per h, respectively, to characterize CEQ in the dialysate. For the current model, the lung compartment was proposed and validated by microdialysis. Finally, the PBPK parameters for the transfer rate were determined by the model fitting.

Given that lung interstitial fluid is the focus of infection by respiratory tract pathogens, the concentration of unbound antimicrobial agent(s) in this compartment is thought to be responsible for antimicrobial efficacy. Microdialysis is a promising technique to sample the unbound drug concentration in the lung. During a microdialysis experiment, it is important to maintain anesthesia. Slight body movement and excessive lung dilation will disturb the microdialysis probe or influence the results. Zoletil and propofol were selected to maintain anesthesia. Heart rate, respiratory rate, and arterial oxygen saturation were monitored. *In vivo* recovery may be different among individuals [31], so the *in vivo* recovery was determined in each piglet and the drug concentration in lung interstitial fluid was corrected. Compared with a previous study (Rottbøll and Friis, 2016), the *in vivo* RRs for different types of microdialysis probes are different in lung. The principle of microdialysis is based on the presence of a concentration gradient between two fluid compartments across a semipermeable membrane. There are some publications about CEQ microdialysis experiments (Rottbøll and Friis, 2016; Zhang et al., 2019c), the AUC_lung interstitial fluid_ to AUC_plasma(fu)_ ratio of cefquinome has been reported as 1.3 in swine, ranged from 0.92 to 1.58. In the current study, the AUC_lung interstitial fluid_ to AUC _plasma_ ratio of cefquinome was 0.86. Individual differences and sampling errors may be the reasons for the difference.

A local sensitivity analysis was performed for all parameters. QCC, QKC, and KurineC are sensitive to the AUCs of almost all tissues. The tissue partition coefficients of lung, muscle, liver, and kidney are highly sensitive to the AUCs of the corresponding tissues but do not influence the other tissues. These findings are not surprising since most of the drug concentration in a tissue is directly related to that tissue’s partition coefficient. The parameters related to the lung, including the chemical-specific parameters KIT, KTI, and PT, are influenced the drug concentrations in the lung interstitial fluid sub-compartment. Bodyweight also influences the AUC of lung interstitial fluid. The PBPK model in this study is based on piglets with a body weight of about 25 kg (these animals are susceptible to respiratory tract pathogens).

Due to high morbidity and mortality, respiratory tract diseases have a substantial economic impact on intensive pig production [32]. Antibiotics represent a major way to treat respiratory tract diseases, but excessive and indiscriminate use facilitates the emergence of antibiotic resistance. In China, from 2015 to 2017, pathogens that cause respiratory tract diseases in swine displayed a rapid increase in antibiotic resistance rates, which may be indicative of abuse and misuse of antibiotics [33,34]. It is important to determine an optimized dosage regimen to guide clinical treatment. It is widely accepted that PK/PD principles can be applied to predict effective dosages of antibacterial drugs in veterinary medicine [10]. PK/PD parameters serve a vital role in evaluating and determining the dosage regimen [35].

CEQ is recommended to treat swine respiratory tract disease based on the excellent characterization of its in vitro and in vivo antibacterial activity. It is important to determine optimized dosage regimens of CEQ to ensure it can produce clinical efficacy. PK/PD parameters are valuable indicators of in vivo efficacy. %T > MIC, the time the unbound concentration exceeds the MIC, is usually selected as the best PK/PD parameter to determine the time-dependent antibacterial activity of a drug such as CEQ. Craig determined the PK/PD parameters of beta-lactams against gram-negative and gram-positive pathogens. %*f*T>MIC is defined as 25%-40% of the dosage regimen for gram-positive pathogens and 40%-50% for gram-negative pathogens is regarded as effect [36]. Toutain et al. have introduced when T>MIC, for beta-lactams, was 40%-50%, the survival rate of infectious animal can reach 90%-100%(Toutain et al., 2002). Papich MG adapted T>MIC of 40% the the target value for cefazolin can achieve antibacterial effect (Papich, 2014). And %T>MIC needs approximately 20%–40% of the dosing interval for beta-lactams to perform bactericidal effect (Turnidge, 1998). In this study, a pop-PBPK model was established and PK/PD parameters were integrated to evaluate the dosage regimen of CEQ against respiratory tract pathogens of swine. As Fig 3 shown, the predicted for the peak of PELF concentration is overestimated. The overestimate may impact on PD estimates. In this manuscript, a pop-PBPK model which can simulate the PK profiles of 1000 animals were developed which involved the coefficient variance among animals. It can reduce the impact by the overestimation.

Monte Carlo analysis is a powerful tool that can generate multiple PK profiles to assess the dosage by comparing with PK/PD parameters [37]. This approach allows assessing inter-individual variability in the sensitive parameters of the PBPK model [38]. This method is mostly used in the framework of toxicological assessment [4,16]. The distribution and variability of sensitive parameters are based on previous investigation. The coefficient variability of physiological parameters, collected by Lindstedt for the ILSI Risk Science Institute Physiological Parameters Working Group, are ranged from 6~30%. Partition coefficient variability was directly measured for perchloroethylene, and CV is ranged of 15~20%. These CV values are well accepted in the field of PBPK modelling and are often used in recently published papers. The current study is the first to assess the PTA of different dosage regimens by PBPK model and PK/PD parameters. PTA is usually used to optimize and justify dosage regimen [13]. After the pop-PBPK simulation, individuals in the 10th percentile who can reach the PK/PD parameters were calculated. As shown in **Table 1** shown, individuals in the 10th percentile can reach the PK/PD parameter of 15.3% in the plasma and 17.6% in the lung interstitial fluid at the MIC of 0.25 μg/mL, and 8.13% in the plasma and 9.05% in the lung interstitial fluid at the MIC of 1 μg/mL. These values indicate an ineffective antibacterial effect. At the extra-label dose of 3 mg/kg delivered twice daily, the PK/PD parameters exceeded the target value, except for *S*. *suis*, which is not susceptible to CEQ. Note, these doses are designed for the 25 kg piglets, for the market-age swine (90 kg), the dose need to be re-assessed.

In addition, pop-PBPK models are widely used to estimate the WDIs of label and extra-label dosage regimens [39]. In this model, the withdrawal time was estimated using the pop-PBPK model to simulate the concentration-time profiles of 1000 virtual animals. A sensitive parameter is assumed to have a distribution within the lower and upper bounds, which are calculated by using 95% confidence intervals [40]. Generally, different country or regulatory jurisdictions may adopt different marker residues and MRL/tolerance. The MRLs for cefquinome of 0.1 and 0.2 PPM in liver and kidney, respectively are both used in China and EU [23], while the FDA does not set the tolerance of cefquinome. At the label dosage (2 mg/kg once daily), the WDI was calculated as 2 days. A previous study reported a WDI of 3 days in swine, which is conservative compared with the present study. High variability in the model may underscore the different estimates.

There are some limitations in the manuscript. This PBPK model is established for the swine with bodyweight of 25 kg. With the bodyweight changed, the PK profiles of cefquinome in PELF, serum and edible tissues are also changed. For the higher bodyweight, as 100 kg, the dosage regimen and withdrawal intervals determined in this model are not applicable. The dataset used for model validation is sparse. More data in liver and kidney about the residual elimination are needed that can make the prediction in edible tissues more accurate. For the WDI estimation, the drug concentration in the last-time point is important. Of note, in the current study only sensitive parameters were defined as those with different distributions. And the CV of model parameters need to be set more reasonably.

## Materials and methods

### Ethics statement

The research was approved by the Ethics Committee of the Faculty of Veterinary Medicine of the Huazhong Agricultural University with the ethics number is 202109140008. All animal experiments were conducted according to the guidelines of the Laboratory Animal Use and Care Committee at Hubei Science and Technology Agency.

### Chemicals and reagents

A CEQ standard (Dr. Ehrenstorfer Standards, Augsburg, Germany) was used for ultra-performance liquid chromatography–tandem mass spectrometry (UPLC-MS/MS). Cefquinome sulfate injection was purchased from Amicogen Biopharm Co. Ltd. (Jining, China). Ringer’s solution was purchased from Yuanye Bio-Technology Co., Ltd. (Shanghai, China).

### Animals

Four crossbred pigs (Landrace × Large White × Duroc) weighing 20 ± 2 kg were purchased from Jinlin Swine Genetics, Ltd. (Hubei, China).

### *In vitro* calibration of microdialysis

To ensure the microdialysis probe can convert the *in vivo* drug concentration to the tissue concentration, the proportion of drug diffusion over the microdialysis membrane (recovery) must be established. Calibration involves determining the *in vitro* relative recovery (RR), delivery, and recovery by applying Eqs 1 and 2.


$$Recovery(\%)=100\times \frac{C_{dialysate}}{C_{medium}}$$ (1)

$$Delivery(\%)=100\times (1-\frac{C_{dialysate}}{C_{perfusate}})$$ (2)

In the above equations, C_dialysate_ is the concentration of CEQ in the dialysate, C_medium_ is the CEQ concentration in Ringer’s dilution, and C_perfusate_ is the CEQ concentration in the perfusate. Different drug concentrations (0.5, 1, 2.5, and 5 μg/mL) in Ringer’s solution and different flow rates (1, 1.5, and 2 μL/min) were applied. Each experiment was repeated three times. The optimized flow rate was used to determine the *in vivo* RR.

### PK experiment

#### Probe implantation and sample collection

The microdialysis probe was implanted as described previously [41]. Briefly, 0.05 mg/kg of atropine sulfate (Shanxi Ruicheng Kelong Veterinary Medicine Co., Ltd., China) was administrated intramuscularly and 5 mg/kg Zoletil 50 (Vibrac, Carros, France) were injected intramuscularly to induce anesthesia. The animals were placed in the supine position and intubated with a tracheal tube (internal diameter 5–6 cm) to connect to the ventilator. Positive end-expiratory pressure and the recruitment maneuver were performed as described previously [42] to protect the lung from ventilator-induced lung trauma. Anesthesia was maintained by Zoletil 50 with 2 mg/kg/h and propofol (Xian Libang Pharmaceutical Co., Ltd., China) with 5 mg/kg/h. One rib (6th-7th) was removed for better exposure to implant the microdialysis probe. The introducer needle was inserted horizontally through the lower lobe of the left lung. The CMA/30 probe (CMA Microdialysis AB, Solna, Sweden) was inserted through this introducer needle, which was then removed.

After implantation, the microdialysis probe was allowed to acclimate for 60 min while being perfused continuously with Ringer’s dilution containing 0.75 μg/mL CEQ delivered at 1 μL/min by a CMA/402 pump controller (CMA Microdialysis AB). The *in vivo* calibration was performed by retrodialysis for each animal at 1 μL/min for 60 min. The probe *in vivo* RR was determined by comparing the “input” (perfusate) and “output” (dialysate) concentrations (Eq 3).


$$In\;vivo\;RR(\%)=100\times (\frac{C_{perfusate}-C_{dialysate}}{C_{perfusate}})$$ (3)

The CEQ concentration in the interstitial fluid of each lung was calculated as: C_ISF_ = C_dialysate_ / *In vivo* RR.

After a 60-min wash-out period (microdialysis system), CEQ was injected intramuscularly at 2 mg/kg body weight. Dialysates were sampled at 0.25, 0.5, 0.75, 1, 1.25, 1.75, 2.25, 3.25, 4.25, 5.25, 7.25 9.25, 10.25, and 11.25 h after drug administration. The samples were stored at -20°C until analysis. During microdialysis, the body temperature, arterial blood oxygen saturation, heart rate, and respiratory rate were monitored to ensure the state of the pig.

### UPLC-MS/MS

The CEQ concentration in different samples were determined by Waters Acquity UPLC-MS following a published protocol [43]. Chromatographic separation was achieved on an Acquity BEH C_18_ column (100 mm × 2.1 mm × 1.7 μm) at 40°C. The mobile phase consisted of solution A (water with 0.1% formic acid, v/v) and solution B (acetonitrile) at a flow rate of 0.3 μL/min. The gradient elution was: 1–4 min, 5%-50% B solution; 7.5–7.6 min, 70%~5% B; 7.6–9 min, 5% B. The injection volume was 10 μL. The dialysate sample was diluted 10-fold by the mobile phase and then used for the test. The CEQ concentration in the dialysate was determined by the standard curve of the dialysate. The equation was y = 39305x – 1482.4 (coefficient of determination [R^2^] = 0.9993) within the concentration range of 0.05–10 μg/mL. The lower limit of determination (LLOD) and the limit of quantitation (LLOQ) were 0.02 and 0.05 μg/mL, respectively.

### Data sources for model calibration and validation

The PK data used for the calibration and validation of the PBPK model are summarized in **Table 2.** The CEQ plasma concentrations are from published studies and extracted from WebPlotDigitizer (version 4.4, https://automeris.io/WebPlotDigitizer/). All data were derived from healthy animals.

**Table 2:** Published cefquinome pharmacokinetic studies used for calibration and validation of the physiologically based pharmacokinetic model.

PK study and purpose	Route^a^	n	Body weight(kg)^b^	Dose regimen	Dose (mg/kg)	Tissue
***Calibration***						
Li, Wu [19]	IM	5	25	Single injection	2	Plasma
Zhang, Li [18]	IM	40	45	5 doses over 24 h	2	Liver/kidney
Experiment^d^	IM	3	20	Single injection	2	Lung interstitial fluid
***Validation***						
Mi, Li [17]	IM	6	15	Single injection	2	Plasma
Xu., Yang. [21]^c^	IM	40	30	5 doses over 24 h	2	Liver/kidney
Experiment^d^	IM	1	20	Single injection	2	Lung interstitial fluid

^a^ IM, intramuscular injection

^b^ The average bodyweight in the experiment.

^c^The cefquinome concentrations in different tissues for the model validation are shown in **S1 Table**

^d^ The concentration-time profile of cefquinome in lung interstitial fluid was determined by microdialysis

### Model structure

**Fig 6:**
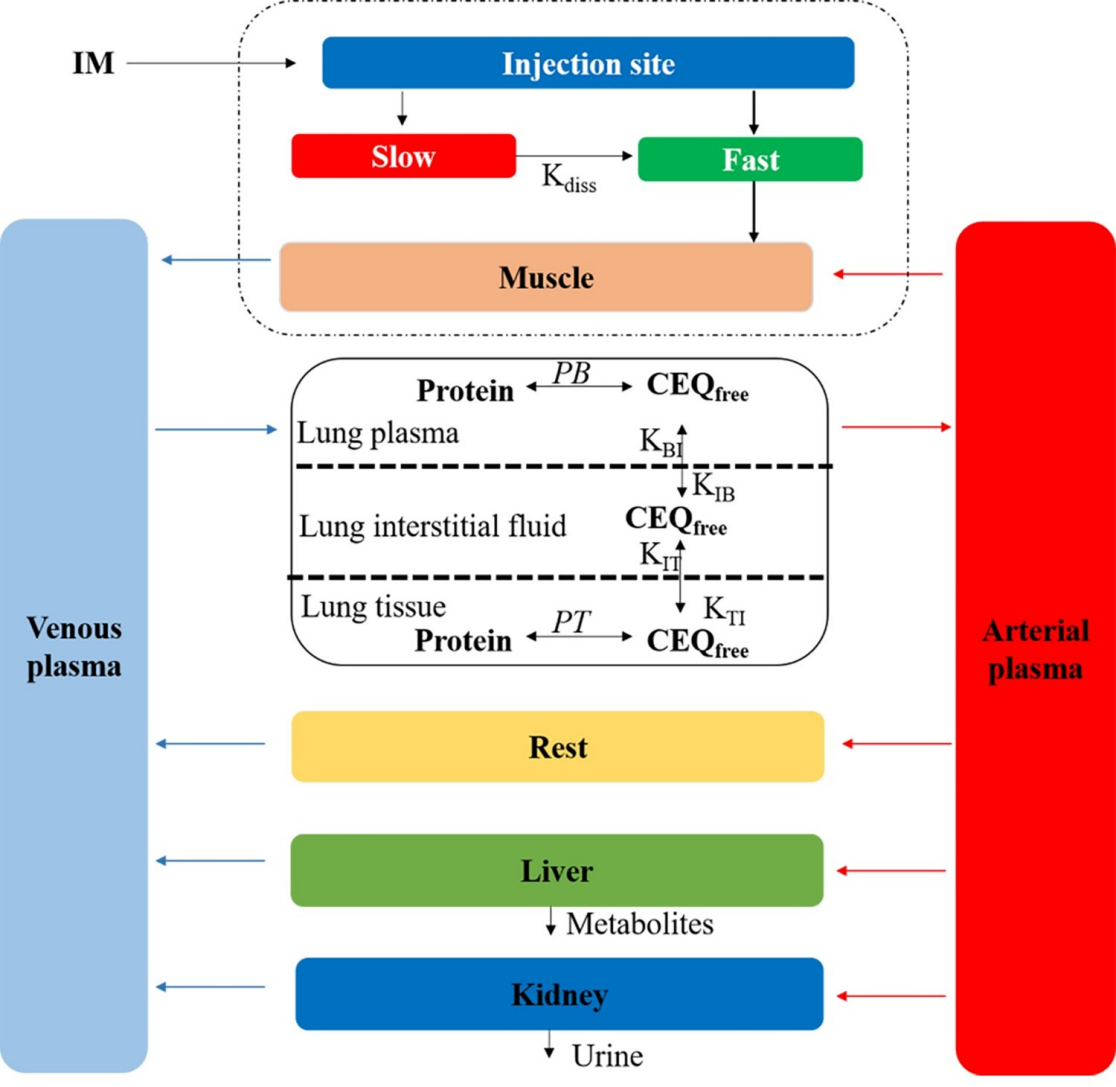
A schematic diagram of the physiologically based pharmacokinetic model for cefquinome in swine.

The PBPK model of CEQ in swine was established based on previous studies [8,44]. A six-compartment PBPK model, including plasma, liver, kidney, lung, muscle, and the rest of the organs, connected by the blood circulation, is shown in **Fig 6**. Each compartment is defined by a tissue weight and tissue blood flow rate. All compartments (except lung) are assumed to be perfusion-limited and well stirred. The kinetic of cefquinome in lung is assumed as permeability-limited and lung compartment comprise three sub-compartments, namely lung blood, lung interstitial fluid, and lung tissue. The present PBPK model was developed based on Berkeley Madonna (Version 10.1.3).

A two-compartment model is used to describe drug absorption into muscle after intramuscular injection [20]. As shown in **Fig 6**, the injection bolus is assumed to be absorbed in two steps, fast and slow. Fast absorption means that the drug homogeneously mixes at the injection site and is instantaneously available for absorption into the venous compartment with a first-order process (k_im_) [45]. Slow absorption refers to CEQ release from the injection site as a first-order rate (*k*_*diss*_) and entering the venous circulation as a fast phase.

According to the European medicine agency (EMA), CEQ is mainly excreted by the kidney and liver. The current model also includes renal clearance (KurineC) and hepatic clearance (KbileC) are also described. CEQ is an organic acid with pKa values of 2.51 or 2.91, and its distribution in the body is extensive [23]. CEQ is not detectable in muscle (non-injection site) after continuous intramuscular injection based on a radioligand study [1] and a residue depletion study [18]. The concentration-time profiles of CEQ in muscle were not simulated in this study.

For the lung sub-compartment, unbound CEQ can be transported between the plasma, interstitial fluid, and lung tissue. Protein binding rates for different matrixes are different, which are parameterized as *PB* for blood and *PT* for lung tissue. The free fraction of CEQ in the interstitial fluid can be directly determined by microdialysis. The free drug can cross the sub-compartments at a first-order rate driven by the drug concentration.

### Model parameterization and calibration

The physiological parameters of swine were based on the previous study [29]. The chemical-specific parameters, such as tissue/plasma partition coefficient, were used as initial values which was calculated from the previous [46] and the “final model parameters” need to be adjusted and optimized by comparing simulations and observed PK data. PBPK model is only calibrated by the calibration data which were listed in **Table 2**. And the values of all parameters used in the PBPK model are shown in **Table 3.**

**Table 3:** Physiological parameters of swine and chemical-specific parameters of cefquinome used in the physiologically based pharmacokinetic model.

Parameter (unit)	Abbreviation	Mean	Reference
Bodyweight (kg)	BW	25	[29]
Cardiac output (L/h/kg)	QCC	4.944	[29]
**Organ blood flow (% of QCC)**			
Muscle	QMC	0.2524	[29]
Rest	QRC	0.3055	Calculated
Liver	QLC	0.3053	[29]
Kidney	QKC	0.1398	[29]
Lung	QLUC	1	[29]
**Organ volume (% of BW)**			
Lung	VLUC	0.01	[29]
Muscle	VMC	0.4	[29]
Rest	VRC	0.232	Calculated
Liver	VLC	0.0294	[29]
Kidney	VKC	0.004	[29]
Arterial blood	VartC	0.016	[29]
Venous blood	VvenC	0.044	[29]
**Tissue to blood partition coefficient**			
Lung	PLU	1.5	[46]
Muscle	PM	0.1	Model fitting
Rest	PR	0.1	Model fitting
Liver	PL	6	[46]
Kidney	PK	15.2	[46]
**Absorption rate constant (/h)**			
	Kim	7	Model fitting
	Frac	0.1	Model fitting
	Kdiss	0.05	Model fitting
Hepatic clearance (L/h/kg)	KbileC	0.01	Model fitting
Renal clearance (L/h/kg)	KurineC	0.3	Model fitting
Percentage of plasma protein binding	PB	0.188	[31]
Percentage of lung tissue protein binding	PT	0.3	Model fitting
**Sub-compartment volume (% of lung)**			
Lung Blood	VLUB	0.262	[30]
Lung interstitial fluid (IF)	VLUI	0.188	[8]
Lung tissue	VLUT	0.55	Calculated
The constant rate of blood to IF (/h)	KBI	0.110	Model fitting
The constant rate of IF to blood (/h)	KIB	0.052	Model fitting
The constant rate of IF to tissue (/h)	KIT	3.56	Model fitting
The constant rate of tissue to IF (/h)	KTI	2.60	Model fitting

### Model validation

The current model was validated by comparing the results of previously available data (as **Table 2** shown). Based on World Health Organization (WHO) guidelines, model simulations are available within a twofold range of the measured values [47]. The goodness of fit between the simulation and observations was analyzed with linear regression for both calibration and validation; R^2^ was calculated. R^2^ ≥ 0.75 is regarded as a general criterion for good prediction [4]. The mean absolute percentage error (MAPE) was also used to validate the model [15]. The validation criteria of MAPE are: (i) excellent prediction: MAPE < 10%; (ii) good prediction: 10% < MAPE < 20%; and (iii) acceptable prediction: MAPE < 50%. A MAPE value < 50% was considered to be an acceptable prediction [5].

### Sensitivity analysis

A local sensitivity analysis was performed for a discrete time point (24 h) to determine the most influential parameters on the area under the curves (AUCs) for the plasma, liver, kidney, and lung (including lung blood and lung interstitial fluid) CEQ concentrations. By increasing each parameter by 10%, the AUC of different tissues can be simulated. The normalized sensitivity coefficient (NSC) was calculated using Eq 4.


$$NSC=\frac{\Delta r}{r}\times \frac{p}{\Delta p}$$ (4)

In this equation, p is the original parameter value, Δp is the change from the original parameter (p / Δp = 10%), r is the original output result, and Δr is the change in the model output resulting from altering the parameter. A parameter was considered influential if the absolute value of its NSC was > 0.25.

### Pop-PBPK model

Monte Carlo analysis was added to the PBPK model to estimate the effects of parameter uncertainty and individual variability. The parameters were randomly selected from the specific parameter distribution around the mean and within the 95% confidence interval for each iteration. Only the influential parameters are simulated by Monte Carlo analysis. Physiological parameters are generally considered to have a normal distribution, and chemical-specific parameters are considered to have a lognormal distribution. The default coefficient of variation (CV) for partition coefficients (PCs) and transport constant rates are assumed to be 20%. The default CVs of other parameters were set as 30%. The lower bound (2.5 percentile) and upper bound (97.5 percentile) of the distribution were also set to constrain the range of random selection. **Table 4** presented the distributions of specific parameters including mean, standard deviate, upper bound, and lower bound.

**Table 4:** Values and distributions of parameters in the Monte Carlo analysis for the physiologically based pharmacokinetic model.

Parameter	Distribution	Mean	CV	SD	Lower	Upper
QCC	Normal	4.944	0.3	1.48	2.04	7.85
QKC	Normal	0.1398	0.3	0.04	0.06	0.22
PL	Lognormal	6.00	0.20	1.20	3.99	8.67
PK	Lognormal	15.2	0.20	3.04	10.11	21.97
PM	Lognormal	0.10	0.20	0.02	0.07	0.14
PR	Lognormal	0.10	0.20	0.02	0.07	0.14
PLU	Lognormal	1.50	0.20	0.30	1.00	2.17
KIT	Lognormal	3.56	0.30	1.07	1.92	6.06
KTI	Lognormal	2.60	0.30	0.78	1.40	4.43
KurineC	Lognormal	0.30	0.30	0.09	0.16	0.51
PT	Lognormal	0.30	0.30	0.09	0.16	0.51

Note: See Table 3 for a description of the parameters. CV, coefficient of variation; SD, standard deviation

### Model application

The Monte Carlo analysis was set up to run 1000 times with model parameters randomly selected from the defined distributions. It can provide concentration-time profiles for each of the 1000 individuals. The median, 10th, and 90th (99th for the WDI estimation) percentiles of simulated results were calculated and plotted without confidence intervals.

### Dosage validation

The PK/PD parameter is an essential value to assess and determine the dosage regimen. %T > MIC is considered to be a good indicator correlated with *in vivo* drug efficacy for β-lactam antibiotics [36]. For β-lactam antibiotics, %T > MIC is defined as 25%-40% of the dosage regimen for gram-positive pathogens and 40%-50% for gram-negative pathogens to indicate a high likelihood of success [22]. Based on previous studies, the MIC against *H*. *parasuis* and *S*. *suis* as was set at 1 μg/mL, whichi was the clinical breakpoint of CEQ against *H*. *parasuis* and *S*. *suis* [17,48]. A MIC of 0.25 μg/mL were set as a reference value to judge the susceptibility change for *P*. *multocida* and APP [49].

T > MIC was determined by using the conditional operator “IF … THEN … ELSE …” to create a controlling factor. If the free drug concentration surpasses the MIC, 0 is assigned to the control factor. In that case, no additional time will be accounted into the model. Eq 5 shows an example of the T > MIC calculation:

$$\begin{array}{l} \mathrm{xTime}=\mathrm{if}\;\mathrm{CAfree}>1\\ \mathrm{then}\;1\;\mathrm{else}\;0\\ d/\mathrm{dt}(\mathrm{TimeaboveMIC})=\mathrm{xTime}\\ \mathrm{init}\;\mathrm{TimeaboveMIC}=0 \end{array}$$ (5)

where xTime represents the control factor, CA_free_ is the unbound drug concentrations in the arterial compartment, and TimeaboveMIC is the PK/PD parameter (T > MIC). T > MIC is divided by the administration interval to derive %T > MIC.

By inputting different dosages into the PBPK model, the sensitivity parameter was set as a distribution (normal or lognormal) within the 95% confidence intervals to perform the Monte Carlo analysis. The concentration-time profiles and PK/PD parameters (%T > MIC) in plasma and lung interstitial fluid were simulated for 1000 virtual individuals. PTA was determined by simulation with the pop-PBPK model using different dosage regimens. Comparing PTA and the recommended PK/PD parameters can reveal an effective dosage regimen.

### WDI estimation

The MRLs were only determined by the European Union and China, which is 0.1 PPM in the liver and 0.2 PPM in the kidney. The WDIs of label and extra-label dosages can be predicted by a pop-PBPK model. The WDI was determined when the 99th percentile tolerance of tissue residues falls within the MRL [50].

## Supporting information

S1 Fig The result of MAPE. (DOCX)

S1 Table Validation dataset of PBPK model. Residue concentration of cefquinome in tissues after five intramuscular injections at dose of 2mg/kg with 24 intervals
(DOCX)

S2 Table The results of sensitive analysis. Normalized sensitivity coefficients (NSCs) of sensitive parameters on the area under the concentrations (AUCs) of cefquinome in plasma, liver, kidney, muscle, rest, lung and lung interstitial fluid.
(DOCX)

S1 Text PBPK Code of cefquinome in swine. (DOCX)
