# Supplementary material for: A physiologically based pharmacokinetic model to optimize the dosage regimen and withdrawal time of cefquinome in pigs
Source: PLoS Comput Biol. 2023 Aug 16;19(8):e1011331. doi: 10.1371/journal.pcbi.1011331 (PMC10431683; doi:10.1371/journal.pcbi.1011331)
Supplement: S1 Fig — (DOCX) [file pcbi.1011331.s001.docx]

## S1 Fig. The result of MAPE.


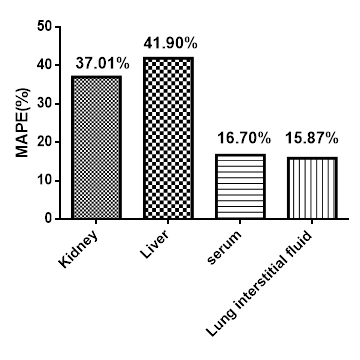


**S1 Fig.** **The mean absolute percentage error (MAPE) analysis for results of the model evaluation.**
