# Supplementary material for: A physiologically based pharmacokinetic model to optimize the dosage regimen and withdrawal time of cefquinome in pigs
Source: PLoS Comput Biol. 2023 Aug 16;19(8):e1011331. doi: 10.1371/journal.pcbi.1011331 (PMC10431683; doi:10.1371/journal.pcbi.1011331)
Supplement: S1 Table — Residue concentration of cefquinome in tissues after five intramuscular injections at dose of 2mg/kg with 24 intervals (DOCX) [file pcbi.1011331.s002.docx]

## S1 Table. Validation dataset of PBPK model.

S1 Table. Residue concentration of cefquinome in tissues after five intramuscular injections at dose of 2mg/kg with 24 intervals (n=5).

| Withdrawal days | Concentration (PPB) | | | |
| --- | --- | --- | --- | --- |
|  | Muscle | Fat | Liver | Kidney |
| 0.5 | <LOD | <LOD | 165.01±69.12 | 381.62±187.93 |
| 1 | <LOD | <LOD | 67.59±21.43 | <LOD |
| 2 | <LOD | <LOD | <LOD | <LOD |
| 3 | <LOD | <LOD | <LOD | <LOD |
| 5 | <LOD | <LOD | <LOD | <LOD |
| 7 | <LOD | <LOD | <LOD | <LOD |
