# Supplementary material for: A physiologically based pharmacokinetic model to optimize the dosage regimen and withdrawal time of cefquinome in pigs
Source: PLoS Comput Biol. 2023 Aug 16;19(8):e1011331. doi: 10.1371/journal.pcbi.1011331 (PMC10431683; doi:10.1371/journal.pcbi.1011331)
Supplement: S2 Table — Normalized sensitivity coefficients (NSCs) of sensitive parameters on the area under the concentrations (AUCs) of cefquinome in plasma, liver, kidney, muscle, rest, lung and lung interstitial fluid. (DOCX) [file pcbi.1011331.s003.docx]

## S2 Table. The results of sensitive analysis.

S2 Table. **Normalized sensitivity coefficients (NSCs) of sensitive parameters on the area under the concentrations (AUCs) of cefquinome in plasma, liver, kidney, muscle, rest, lung and lung interstitial fluid.** AUCCV,AUCCK, AUCCM, AUCCR, AUCCLU, AUCCLUI, AUCCL is the area under concentration of serum, kidney, muscle, rest of body, lung, lung interstitial fluid and liver.

| Sensitive Parameters | AUCCV | AUCCK | AUCCM | AUCCR | AUCCLU | AUCCLUI | AUCCL |
| --- | --- | --- | --- | --- | --- | --- | --- |
| QCC | -0.29 | 0.01 | -0.29 | -0.29 | -0.29 | -0.29 | -0.28 |
| QKC | -0.26 | 0.01 | -0.26 | -0.26 | -0.26 | -0.26 | -0.26 |
| PL | 0.00 | 0.00 | 0.00 | 0.00 | 0.00 | 0.00 | 1.00 |
| PK | 0.00 | 1.00 | 0.00 | 0.00 | 0.00 | 0.00 | 0.00 |
| PM | 0.00 | 0.00 | 1.00 | 0.00 | 0.00 | 0.00 | 0.00 |
| PR | 0.00 | 0.00 | 0.00 | 1.00 | 0.00 | 0.00 | 0.00 |
| PLU | 0.00 | 0.00 | 0.00 | 0.00 | 1.00 | 1.00 | 0.00 |
| PT | 0.00 | 0.00 | 0.00 | 0.00 | 0.00 | -0.31 | 0.00 |
| KIT | 0.00 | 0.00 | 0.00 | 0.00 | 0.00 | -0.67 | 0.00 |
| KTI | 0.00 | 0.00 | 0.00 | 0.00 | 0.00 | 0.70 | 0.00 |
| KurineC | -0.60 | -0.88 | -0.60 | -0.60 | -0.60 | -0.60 | -0.60 |
