# Supplementary material for: A physiologically based pharmacokinetic model to optimize the dosage regimen and withdrawal time of cefquinome in pigs
Source: PLoS Comput Biol. 2023 Aug 16;19(8):e1011331. doi: 10.1371/journal.pcbi.1011331 (PMC10431683; doi:10.1371/journal.pcbi.1011331)
Supplement: S1 Text — (DOCX) [file pcbi.1011331.s004.docx]

S1 Text. PBPK Code of cefquinome in swine.

**; Berkeley Madonna Code of cefquinome in swine**

METHOD RK4

STARTTIME = 0

STOPTIME=24 ; h, 24

DT = 0.001

DTOUT = 0.01

{Physiological Parameters}

; Blood Flow Rates

QCC = 4.944 ; L/h/kg, Cardiac Output

; Fracion of blood flow to organs (unitless)

QLC = 0.3053 ; Fraction of flow to the liver

QKC = 0.1398 ; Fraction of flow to the kidneys

QMC = 0.2524 ; Fraction of flow to the muscle

QRC= 1 -QLC -QKC- QMC; Fraction of flow to the rest

QLUC= 1

; Tissue Volumes

BW = 25 ; Body weight(kg)

VLC = 0.0294 ; Fractional liver tissue

VKC = 0.004 ; Fractional kidney tissue

VMC = 0.4 ; Fractional muscle tissue

VbloodC = 0.06 ; Blood volume, fractional of BW

VvenC = 0.044 ; Venous blood volume, fraction of BW

VartC = 0.016 ;Arterial blood volume, fraction of blood BW

VLUC = 0.01 ; lung volume, fraction of blood BW

VRC=1-VLC-VKC-VMC-VbloodC-VLUC ; the rest volume, fraction of blood volume

{Mass Transfer Parameters (Chemical-Specific Parameters)}

; Partition Coefficients (PC, tissue:plasma)

PL = 6; Liver: plasma PC

PK =15.2; Kidney:plasma PC

PM = 0.1; Muscle:plasma PC

PR= 0.1; carcass:plasma PC

PLU=1.5 ;lung:plasma PC

{Kinetic Constants}

; IM Absorption Rate Constants

Kim = 7 ; /h, IM absorption rate constant

Frac = 0.1 ;

Kdiss = 0.05 ;/h

; Percentage Plasma Protein Binding unitless

PB=0.188 ; Percentage of drug bound to plasma proteins

PT= 0.3; Percentage of drug bound to lung proteins

; Fraction of tissue volumes

FVBLU = 0.262; Blood volume fraction of lung (%)

FVILU = 0.188; Blood volume fraction of Interstitial fluid (%)

;Permeability constatns (/h)

KBI =0.110; The constant rate of blood to IF

KIB = 0.052; The constant rate of IF to blood

KIT = 3.56; The constant rate of IF to tissue

KTI = 2.6; The constant rate of tissue to IF

{Metabolic Rate Constant}

; Bile elimination rate constant adjusted by bodyweight

KbileC=0.01 ; L/h/kg

; Urinary Elimination Rate Constants

KurineC = 0.3 ; L/h/kg

{Parameters for Various Exposure Scenarios}

PDOSEim = 2; (mg/kg)

{Cardiac output and blood flow to tissues (L/h)}

QC = QCC * BW; Cardiac output

QL = QLC * QC ; Liver blood flow rate

QK = QKC * QC; Kidney blood flow rate

QM = QMC * QC ; Muscle blood flow rate

QR= QRC * QC ; rest blood flow rate

QLU= QLUC * QC

; Tissue volumes (L)

VL = VLC * BW ; Liver

VK = VKC * BW ; Kidney

VM = VMC * BW; Muscle

Vblood = VbloodC * BW; Blood

VLU=VLUC*BW ;LUNG

VR = VRC*BW ;rest

Vven = VvenC*BW ; Venous Blood

Vart = VartC*BW ; Arterial Blood

;Volume of lung

VLUB = VLU * FVBLU

VLUI = VLU * FVILU

VLUT= VLU-VLUI-VLUB

; BILE elimination rate constant

Kbile = KbileC * BW ; L/h

;Urinary elimination rate constant

Kurine = KurineC * BW ;L/h

; Dosing

DOSEim = PDOSEim * BW ; (mg)

; Dosing, repeated doses

tinterval = 24 ; Varied dependent on the exposure paradigm (h)

Tdoses =5 ; The number of injections for multiple IM

dosingperiod = if time < Tdoses*tinterval-DT then 1 else 0

; Dosing, IM, intramuscular

Rinputim = pulse(DOSEim,0,tinterval)*dosingperiod

Rpenim = Rinputim*(1-Frac);

Rppgim = Rinputim*Frac;

Rim = Kim*Amtsiteim

d/dt(Absorbim) = Rim

init Absorbim = 0

d/dt(Amtsiteim) = Rpenim- Rim + Kdiss* DOSEppgim

init Amtsiteim = 0

d/dt(DOSEppgim) = Rppgim-Kdiss* DOSEppgim

init DOSEppgim = 0

{CEQ distribution in each compartment}

; CEQ in venous blood compartment

RV = (QL*CVL+QK*CVK+ QM*CVM+QR*CVR+Rim)-QC*CV ;

d/dt(AV) = RV ; AV the amount of the drug in the venous blood (mg)

init AV = 0

CV = AV/Vven ; CV drug concentration in the venous blood (mg/L)

RA = QC*(CVLu-CAfree) ; RA the changing rate in the arterial blood (mg/h)

d/dt(AA) = RA

init AA = 0 ; AA the amount of the drug in the arterial blood (mg)

CA = AA/Vart ; CAfree concentration of unbound drug in the arterial blood (mg/L)

CAfree = CA*(1-PB)

d/dt(AUCCV) = CV ; AUCCV AUC of drug concentration in the venous blood (mg*h/L)

init AUCCV = 0

ABlood = AA+AV

; CEQ in liver compartment, flow-limited model

RL = QL*(CAfree-CVL)-Rmet ; RL the changing rate of the amount of drug in liver (mg/h)

d/dt(AL) = RL ; AL amount of drug in liver (mg)

init AL = 0

CL = AL/VL ; CL drug concentration in liver (mg/L)

CVL= AL/(VL*PL) ; CVL drug concentration in venous blood from liver (mg/L)

d/dt(AUCCL) = CL ; AUCCL AUC of drug concentration in liver (mg*h/L)

init AUCCL = 0

; Metabolism of CEQ in liver compartment

Rmet = Kbile * CVL ; Rmet the metabolic rate in liver (mg/h)

d/dt(Amet) = Rmet ; Amet the amount of drug metabolized in liver (mg)

init Amet = 0

; CEQ in kidney compartment, flow-limited model

RK = QK*(CAfree-CVK)-Rurine ; RK the changing rate of the amount of drug in kidney (mg/h)

d/dt(AK) = RK ; AK amount of drug in kidney (mg)

init AK = 0;

CK = AK/VK ; CK drug concentration in kidney (mg/L)

CVK = AK/(VK*PK);

d/dt(AUCCK) = CK ; AUCCK AUC of drug concentration in kidney (mg*h/L)

init AUCCK = 0

; CEQ urinary excretion

Rurine = Kurine*CVK

d/dt(Aurine) = Rurine

init Aurine = 0

; CEQ in muscle compartment, flow-limited model

RM = QM*(CAfree-CVM) ; RM the changing rate of the amount of drug in muscle (mg/h)

d/dt(AM) = RM ; AM amount of the drug in muscle (mg)

init AM = 0

CM = AM/VM ; CM drug concentration in muscle (mg/L)

CVM = AM/(VM*PM)

d/dt(AUCCM) = CM

init AUCCM = 0

; CEQ in the compartment of rest of body, flow-limited model

RR = QR*(CAfree-CVR) ; Rrest the changing rate of the amount of drug in the rest of the body (mg/h)

d/dt(AR) = RR ; Arest amount of the drug in the rest of the body (mg)

init AR = 0

CR = AR/VR ; Crest drug concentration in the rest of the body (mg/L)

CVR = AR/(VR*PR)

d/dt(AUCCR) = CR ; AUCCrest AUC of drug concentration in the rest of the body (mg*h/L)

init AUCCR =0

; CEQ in lung compartment, distribution-limited model

RLUB=QC*(CV-CVLU)-KBI*(ALUB*(1-PB)/VLUB)+(KIB*ALUI/VLUI)

d/dt(ALUB)=RLUB

init ALUB = 0

RLUI=KBI*(ALUB*(1-PB)/VLUB)-(KIB*ALUI/VLUI)-(KIT*ALUI/VLUI)+KTI*(ALUT*(1-PBtissue)/VLUT)

d/dt(ALUI)=RLUI

init ALUI = 0

RLUT=(KIT*ALUI/VLUI)-KTI*(ALUT*(1-PBtissue)/VLUT)

d/dt(ALUT)= RLUT

init ALUT = 0

RBI= KBI*(ALUB*(1-PB)/VLUB)

d/dt(ABI)= RBI

init ABI = 0

CVLU=(ALUI+ALUT+ALUB)/(VLU*PLU)

CLU= (ALUI+ALUT+ALUB)/VLU

d/dt (AUCCLU )= CLU

ALu=CLU*VLU

init AUCCLU = 0

CLUI= ALUI/VLUI

d/dt (AUCCLUI )= CLUI

init AUCCLUI=0

{Mass balance equations}

Qbal = QC-QM-QR- QK-QL

Tmass = ABlood+AM+ALu+AR+AK+AL+Aurine+Amet

Input = Absorbim

Bal = Input-Tmass
